# The mental health of doctors during the COVID-19 pandemic

**DOI:** 10.1192/bjb.2020.44

**Published:** 2020-04-28

**Authors:** Niall Galbraith, David Boyda, Danielle McFeeters, Tariq Hassan

**Affiliations:** 1 Department of Psychology, University of Wolverhampton, UK; 2 Department of Psychiatry, Queen's University, Providence Care Hospital, Kingston, Ontario, Canada

**Keywords:** COVID-19, mental health, coronavirus, healthcare professionals, doctors

## Abstract

Doctors experience high levels of work stress even under normal circumstances, but many would be reluctant to disclose mental health difficulties or seek help for them, with stigma an often-cited reason. The coronavirus disease 2019 (COVID-19) crisis places additional pressure on doctors and on the healthcare system in general and research shows that such pressure brings a greater risk of psychological distress for doctors. For this reason, we argue that the authorities and healthcare executives must show strong leadership and support for doctors and their families during the COVID-19 outbreak and call for efforts to reduce mental health stigma in clinical workplaces. This can be facilitated by deliberately adding ‘healthcare staff mental health support process’ as an ongoing agenda item to high-level management planning meetings.

Research has consistently shown that the healthcare professions experience higher levels of work stress than the general population, even under normal circumstances,^1,2^ and stress in doctors is associated with both physical^3^ and mental health problems.^4,5^ Healthcare professionals also have a higher likelihood of suicidality relative to other occupational groups,^6,7^ and work-related stress is a common factor in those who complete suicide.^8,9^

Studies have also shown that many doctors find it difficult to tell their colleagues or employers about their mental health difficulties.^10^ The most commonly cited reasons are perceived stigma and anticipated damage to future career prospects.^11–13^ Suicidal ideation in doctors can present particularly strong fears of stigmatisation.^14^ Such concerns may be underpinned by feelings of shame and professional failure, and associated worries about fitness to practise and licence restrictions.^15–17^

Not only do doctors find it difficult to share mental health concerns with colleagues, they are also often reluctant to get professional help. Research shows that many doctors would rather seek help from friends and family than look for psychological/psychiatric consultation.^11^ Again, the same worries about career prospects and stigma underpin these preferences. Furthermore, there is evidence that many doctors are even reluctant to disclose mental health problems to their friends and family.^18^

## The mental health challenges faced by doctors during the COVID-19 crisis

During acute health crises, healthcare services are placed under excess pressure, making working life even more stressful than normal.^19^ In a pandemic, the number of patients requiring treatment increases significantly, placing strain on healthcare resources and on personnel alike. Additionally, doctors perceive a greater risk to self owing to their exposure to the patients who are most poorly – adding further stress.^20,21^ Compounding this stress is the shortage of personal protective equipment (PPE) that can arise during a pandemic.^22^ The perceived risk of infection is warranted: a meta-analysis of the occupational risk from the 2009 swine flu pandemic (influenza A (H1N1)) reports that the odds of healthcare personnel contracting the virus were twice those of comparison groups.^23^ This heightened risk for doctors and nurses might be due to their greater exposure to the respiratory secretions of patients.^24^

A further stressor is the increased risk of infection for the families of healthcare professionals on the front line.^25^ Data from the 2009 swine flu pandemic shows that 20% of doctors and nurses with symptoms reported symptoms in at least one of their family members.^26^ One way for front-line doctors to mitigate infection risk to their families is through social distancing. However, although the protective benefits of social contact and support at times of stress are well demonstrated,^27^ social distancing deprives the individual of a crucial buffer against mental health difficulties precisely when they are at greater risk of stress.^28^

Research from previous epidemics/pandemics (such as the SARS outbreak from 2003, the MERS epidemic from 2012 or Ebola outbreaks in West Africa) shows that healthcare professionals can experience a broad range of psychological morbidities, including trauma,^29,30^ which might endure for many months after the outbreak.^31,32^ The relationship between traumatic life events and suicide is well documented^33^ and trauma from disaster events can increase suicidal ideation in emergency workers.^34^ Fears over risk to health and social isolation contribute to psychological distress,^35^ as do perceptions of ‘infection stigma’ from the community.^36^ However, the negative effects on mental health can be found in doctors irrespective of whether or not they worked directly with infected patients.^37^ Although the strains of front-line healthcare during an infectious outbreak can lead to sickness absence and higher staff turn-over,^20,38^ most evidence suggests that doctors and nurses feel a strong professional obligation to continue working in spite of the danger.^39,40^ However, given the pressures of needing to maintain high-quality healthcare provision during a pandemic, combined with doctors’ reluctance to seek help or disclose their difficulties, it is possible that this kind of professional commitment might relate strongly to presenteeism. Indeed, a recent review reported that physicians were at the highest risk of ‘infectious illness presenteeism’ when compared with a range of other occupational groups.^41^

Having to balance their own safety with the needs of patients, family and employers and in the face of limited resources can lead to distressing ethical dilemmas for doctors and, potentially, to moral injury. Moral injury can arise when one feels compelled to make decisions that conflict with one's ethical or moral values.^42^ The effect of moral injury on subsequent mental health can depend on the quality of support provided to employees during and after such events.^43^

## Managing doctors’ stress at the organisational level during the outbreak

There is evidence that employer support for healthcare professionals during pandemics and disaster management can be very protective. Such support should include safeguards such as care for those doctors and nurses who become ill, in addition to medical and financial support for their families and protection from malpractice threat. Healthcare professionals’ motivation and morale are significantly improved when they perceive that their efforts are recognised and reciprocated by employers and authorities in these ways.^44,45^ An important part of this support is the perceived efficacy of the training and personal protective equipment that healthcare professionals receive as well as the general quality of organisational leadership and communication.^22,46,47^ These factors are important not just for motivation – they are also associated with better psychological outcomes in healthcare professionals on the front line during epidemics.

There are also many ways to tackle mental health stigma in the workplace. The foundation for this is creating a culture that encourages open communication and seeks to reduce the stigmatisation of psychological vulnerability.^48^ This may include devising activities that challenge unhelpful attitudes and that instead promote desired values, as well as expanding knowledge and encouraging positive behavioural change.^49^ The anti-stigma project Time to Change,^50^ provides a suite of simple interventions for implementation in the workplace.^51^ Elsewhere there is good evidence for peer support training in health crises or disaster management. One example is the Trauma Risk Management programme (TRiM), where non-clinical personnel are trained to assess peers following traumatic events and provide short-term support or access to professional care if required. Mental Health First Aid (MHFA) operates on a similar model and both it and TRiM can be effective in reducing mental health stigma in the workplace.^45,47^

## Stress management at the individual level

There is evidence that psychological interventions for work stress can be effective in healthcare professionals.^52^ Recent reviews attest to the effectiveness of mindfulness-based interventions for work stress and suicide ideation;^53–57^ mindfulness-based interventions also have a sound theoretical basis.^58,59^ Mindfulness skills are particularly suited to high-stress work settings, in that they can be practised privately or in groups, in almost any environment and can be conducted as briefly as time permits. Negative automatic cognitions are a key trigger in stress reactions.^60^ Mindfulness interventions encourage us to ‘notice’ our thoughts and to view them as objective events that happen *to* us. This enables us to objectify our own negative thoughts, gaining a new perspective on how those thoughts influence our emotions and behaviour and enabling better management of the distress that would normally accompany them. The effectiveness of online mindfulness courses also has a good evidence base.^61^

## Conclusions

Healthcare executives and managers should be aware of the potential for the COVID-19 outbreak to elevate the risk of psychological distress and suicidal ideation in doctors. The literature shows that, although healthcare professionals place high value on provision of training and equipment during such pandemics, effective leadership and managerial support for clinicians and their families are also highly protective against negative psychological outcomes. One of us (T.H.) is involved in setting up a support network of psychiatrists with the sole aim of supporting all physicians during this unprecedented event. Managers and clinicians might also remember that many doctors are reluctant to reveal their difficulties even when experiencing significant psychological distress. Workplace interventions that reduce mental health stigma and promote sharing and support for colleagues with psychological difficulties might improve help-seeking behaviour and attitudes. Mindfulness practice has versatility and a strong evidence base in workplace stress reduction and is therefore a viable technique for groups or individual clinicians to manage stress during the COVID-19 outbreak.

## About the authors

**Niall Galbraith**, Reader in Health Psychology, Department of Psychology, University of Wolverhampton, UK. **David Boyda**, Senior Lecturer in Psychology, Department of Psychology, University of Wolverhampton, UK. **Danielle McFeeters**, Senior Lecturer in Psychology, Department of Psychology, University of Wolverhampton, UK. **Tariq Hassan**, Associate Professor, Department of Psychiatry, Queen's University, Providence Care Hospital, Kingston, Ontario, Canada.

## Supplementary material

For supplementary material accompanying this paper visit http://dx.doi.org/10.1192/bjb.2020.44. click here to view supplementary material
